# The effects of neuron morphology and spatial distribution on the selectivity of dorsal root ganglion stimulation

**DOI:** 10.1088/1741-2552/ad7760

**Published:** 2024-10-15

**Authors:** Juhi Farooqui, Ameya C Nanivadekar, Marco Capogrosso, Scott F Lempka, Lee E Fisher

**Affiliations:** 1 Rehab Neural Engineering Labs, University of Pittsburgh, Pittsburgh, PA 15219, United States of America; 2 Neuroscience Institute, Carnegie Mellon University, Pittsburgh, PA 15213, United States of America; 3 Department of Bioengineering, University of Pittsburgh, Pittsburgh, PA 15213, United States of America; 4 Department of Neurological Surgery, University of Pittsburgh, Pittsburgh, PA 15219, United States of America; 5 Biointerfaces Institute, University of Michigan, Ann Arbor, MI 48109, United States of America; 6 Department of Biomedical Engineering, University of Michigan, Ann Arbor, MI 48109, United States of America; 7 Department of Anesthesiology, University of Michigan, Ann Arbor, MI 48109, United States of America; 8 Department of Physical Medicine & Rehabilitation, University of Pittsburgh, Pittsburgh, PA 15219, United States of America; 9 Department of Biomedical Engineering, Carnegie Mellon University, Pittsburgh, PA 15213, United States of America

**Keywords:** dorsal root ganglion, electrical stimulation, somatosensory restoration, neuromodulation

## Abstract

*Objective.* For prosthesis users, sensory feedback that appears to come from the missing limb can improve function, confidence, and phantom limb pain. Numerous pre-clinical studies have considered stimulation via penetrating microelectrodes at the dorsal root ganglion (DRG) as a potential approach for somatosensory neuroprostheses. However, to develop clinically translatable neuroprosthetic devices, a less invasive approach, such as stimulation via epineural macroelectrodes, would be preferable. This work explores the feasibility of using such electrodes to deliver focal sensory feedback by examining the mechanisms of selective activation in response to stimulation via epineural electrodes compared with penetrating electrodes. *Approach.* We developed computational models of the DRG, representing the biophysical properties of the DRG and surrounding tissue to evaluate neural responses to stimulation via penetrating microelectrodes and epineural macroelectrodes. To assess the role of properties such as neuron morphology and spatial arrangement we designed three models, including one that contained only axons (axon only), one with pseudounipolar neurons arranged randomly (random), and one with pseudounipolar neurons placed according to a realistic spatial distribution (realistic). *Main results.* Our models demonstrate that activation in response to stimulation via epineural electrodes in a realistic model is commonly initiated in the axon initial segment adjacent to the cell body, whereas penetrating electrodes commonly elicit responses in t-junctions and axons. Moreover, we see a wider dynamic range for epineural electrodes compared with penetrating electrodes. This difference appears to be driven by the spatial organization and neuron morphology of the realistic DRG. *Significance.* We demonstrate that the anatomical features of the DRG make it a potentially effective target for epineural stimulation to deliver focal sensations from the limbs. Specifically, we show that epineural stimulation at the DRG can be highly selective thanks to the neuroanatomical arrangement of the DRG, making this a promising approach for future neuroprosthetic development.

## 1. Introduction

Over 3.5 million Americans are projected to be living with limb loss by the year 2050 [1]. Despite advancements in prosthetic technology, most prosthetic limbs still do not provide somatosensory feedback to the user. Somatosensory feedback from the lower limbs is important to locomotion[2], while somatosensory feedback from the fingers and hands is critical for the fine motor control that underlies object manipulation and other desirable functions [3–5]. Neuroprostheses that restore somatosensation after amputation have been shown to improve postural stability [6], gait speed [7], and balance confidence [8] during walking as well as object discrimination and functional grasp improvement [9, 10]. Restored somatosensation can also reduce phantom limb pain [11] and promote prosthetic ease of use [12, 13] and acceptance [14, 15].

Electrical stimulation of neural targets in the somatosensory pathway is a promising method for delivering sensory feedback. While much recent work has targeted the peripheral nerves [6, 7, 16–25] or the spinal cord [26–28], the dorsal root ganglion (DRG) has several potential advantages that make it an attractive target for a neuroprosthesis to restore somatosensation. The DRG are situated lateral to the spinal cord and house the cell bodies of somatosensory afferent neurons projecting from the limbs. Compared with mixed nerves, the DRG represent a relatively clear separation of sensory from motor neurons, reducing the possibility of unintended motor effects from stimulation. Each DRG innervates a single spinal level, and lumbar DRG at a single spinal level can house sensory afferents projecting from a large region of the foot [29]. Additionally, because the DRG are surrounded by the boney neuroforamina of the spine, electrodes implanted nearby may be more stable than those implanted near peripheral nerves which can stretch as the limbs traverse their range of motion [30, 31].

Our lab has performed multiple prior studies in animal models to demonstrate the potential of DRG stimulation as a somatosensory neuroprosthesis [32–35], though these studies have primarily used penetrating microelectrode arrays implanted into the DRG. While the small active contact area of a penetrating electrode gives this approach the potential for high selectivity, penetrating microelectrode arrays create multiple punctures in the tissue and can lead to tissue damage, an elevated immune response to the presence of the device, and instability in the neural interface [34, 36]. Epineural macroelectrodes provide an alternative method for stimulating the DRG, with several potential advantages over penetrating microelectrodes. Because epineural electrodes do not penetrate into the DRG, they are less likely to cause the chronic inflammatory response that degrades the electrode–tissue interface[37]. Moreover, epineural DRG stimulation is a clinically available technology that is approved by the United States Food and Drug Administration for chronic pain management in conditions such as complex regional pain syndrome [38, 39]. As such, there are clinically approved electrodes and established surgical procedures that could be leveraged for delivering sensory feedback. However, these electrodes have contacts with a much larger surface area compared to penetrating electrodes, potentially limiting their selectivity.

A recent pre-clinical study from our lab examined antidromic responses in several peripheral nerve branches in response to stimulation at the DRG via penetrating and epineural electrodes [40]. That study demonstrated that stimulation with an epineural macroelectrode placed on the dorsal surface of the DRG can achieve activation of individual distal nerve branches with comparable selectivity to the activation that can be achieved by stimulation with penetrating microelectrodes. This result was surprising because of the vast difference in electrode surface area and electrode-to-neuron distance between the penetrating and epineural electrodes used in that study. The result is exciting because it suggests that epineural DRG stimulation could be a viable approach to achieve highly selective sensory restoration, but the mechanisms that enable epineural electrode selectivity remain unclear.

Neurons in the DRG have a pseudounipolar morphology [41, 42], consisting of an excitable cell body, which is connected to a stem axon. A relatively long unmyelinated segment of the stem axon adjacent to the cell body is known as the axon initial segment (AIS), which is believed to play a role in action potential initiation and spontaneous activity [43]. The stem axon terminates in a t-junction, where it bifurcates into the dorsal root axon (projecting into the spinal cord) and the peripheral axon (projecting to the limbs). The organization of neurons within the DRG follows a spatial distribution that has been described in literature [44, 45]: cell bodies are more densely packed near the circumference of the DRG, while axons are concentrated in the interior of the structure. The primary goal of this study was to use anatomically and neurophysiologically accurate computational models of the DRG and pseudounipolar afferents to understand the mechanisms that underlie selective activation via epineural and penetrating electrodes in the DRG. To accomplish this goal, we developed realistic computational models of DRG stimulation with penetrating and epineural electrodes. We hypothesized that the presence of large excitable cell bodies near the circumference of the DRG enables selective activation via epineural stimulation, while the high density of axons in the interior of the DRG limits the selectivity of stimulation via penetrating electrodes. A secondary goal of this study was to assess the recruitment of cells of different types in response to stimulation. Since different cell types (e.g. *Aα, Aβ, Aδ*) mediate different sensory modalities (e.g. proprioception, cutaneous sensation, temperature), the ability to selectively activate different cell types in the DRG is also critical for successful implementation of a somatosensory neuroprosthesis.

## 2. Methods

**Figure 1.**
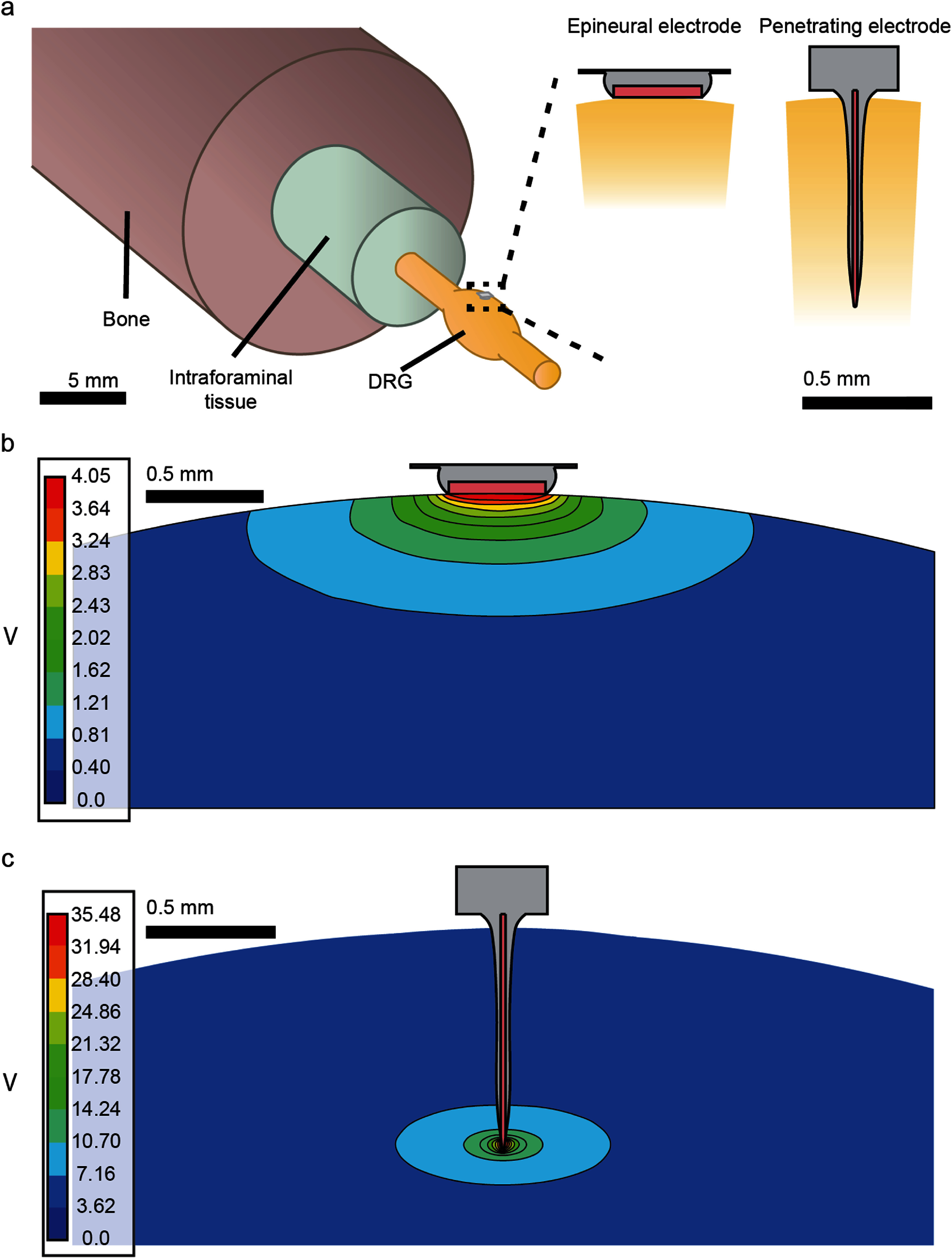
(a) FEM models representing DRG, intraforaminal tissue, bone, and electrodes: epineural (left) and penetrating (right). (b) Epineural electrode placed on the dorsal surface of the DRG and the electric potential field generated by the stimulation. (c) Penetrating electrode inserted through the dorsal surface of the DRG and the electric potential field generated by the stimulation.

**Figure 2.**
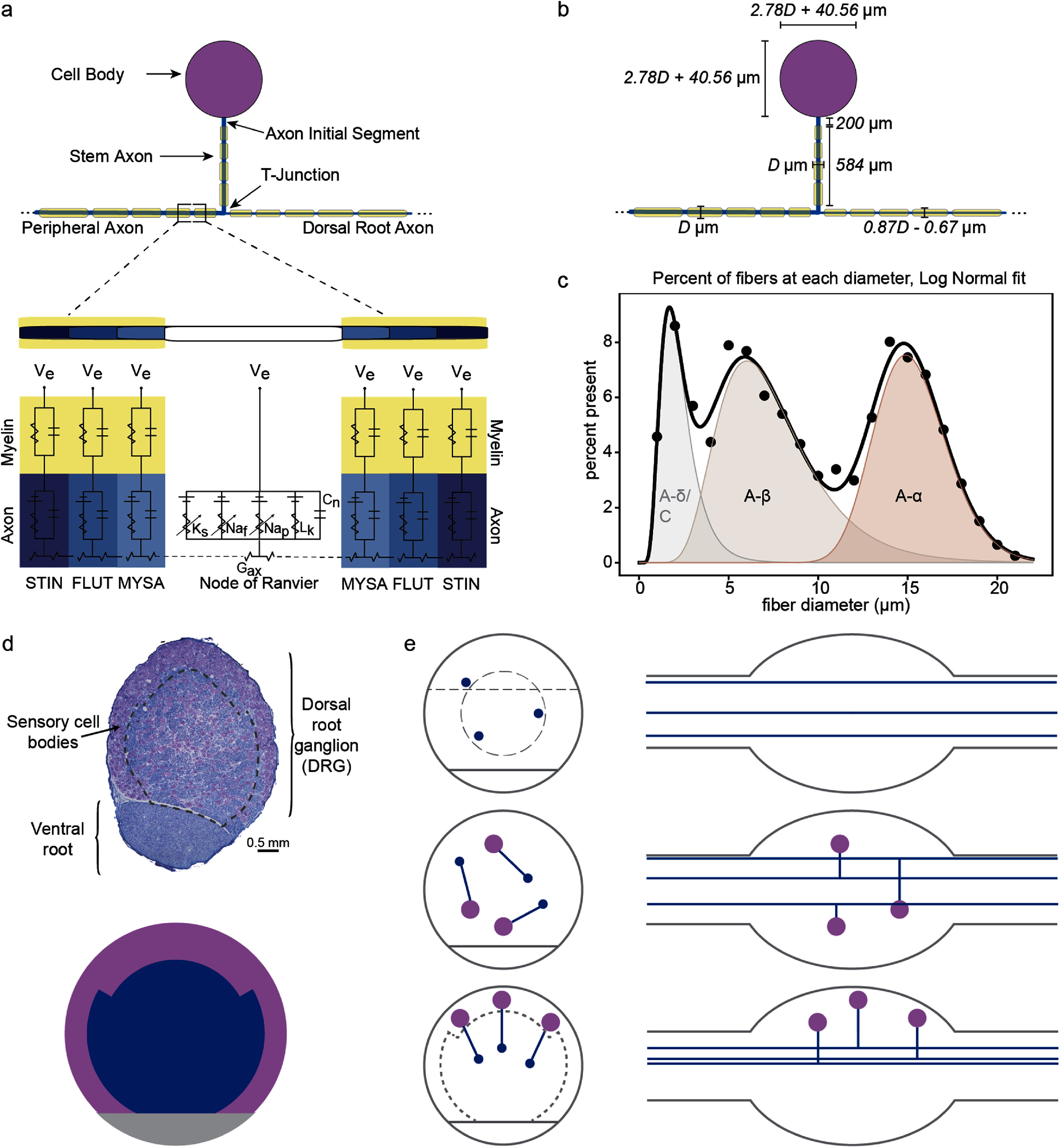
(a) Pseudounipolar neuron morphology and equivalent circuit model of the axon. (b) Sizes of pseudounipolar neuron model sections in relation to fiber diameter. (c) Lognormal distribution of fiber diameters populating the DRG, derived from feline histology data (only *Aα* and *Aβ* neurons were included in this modeling study). (d) Example histology slice (top) showing sensory cell bodies in purple and axons in blue. Dotted line separates outer region containing higher density of cell bodies from inner region containing higher density of axons. Density partition model (bottom). Outer purple region represents the region in which cell bodies are most densely packed. Inner blue region represents a higher density of axons relative to cell bodies. (e) Illustrations of DRG cross-sectional (left) and longitudinal (right) views containing three example neurons (not to scale) representing neuron configurations within DRG: axon-only (top), random (middle), realistic (bottom). Axons of passage are not depicted. Above the dashed line and inside the dashed circle in the top row are the regions from which subsampled neurons were drawn from the axon-only model for the epineural and penetrating electrode simulation respectively, in order to draw from a population with comparable packing density to the realistic model. Outside the dotted line in the bottom row is the region of most densely packed cell bodies in the realistic model (compare with density partition model in panel (d)).

To probe the mechanisms of epineural and penetrating stimulation on DRG neurons, we developed a biophysical computational model of the DRG populated with neuron models representing *Aα* and *Aβ* fiber types. We developed a computational finite element method (FEM) model (figure 1) that replicates the anatomical and electrical properties of the DRG and surrounding tissues as well as the two types of electrodes. We used the FEM to calculate the extracellular electric potentials generated by stimulation of the tissue through epineural and penetrating electrodes, and simulated the dynamics of transmembrane ionic current flow with multi-compartmental models of pseudounipolar neurons (figure 2) placed in this extracellular potential field.

### 2.1. FEM model

The FEM model (figure 1) replicated the anatomical structure of the feline L6 DRG at 0.0001 mm resolution, using measurements taken from previous experiments in feline models. The model included a DRG enlargement modeled as a prolate spheroid with a semi-major axis of 3.7 mm and a semi-minor axis of 1.5 mm, with peripheral and dorsal root branches modeled as 15 cm long cylindrical projections with radius 0.75 mm, attached to the spheroid at 3.25 mm from the center. This structure was ensheathed in a 20 *μ*m thick layer of epineurium and connective tissue, which was surrounded by concentric cylinders representing intraforaminal tissue (3.25 mm radius) and bone (17.2 mm radius). We modeled two types of electrodes, to replicate the electrodes that were used in the previous feline study [40]: (1) a platinum epineural electrode with 375 *µ*m diameter contact placed on the dorsal epineural surface of the DRG and (2) a single-shank Utah array electrode placed at the center of the DRG, penetrating 1 mm into the DRG tissue, with an exposed tip 50 *µ*m in height and 22.5 *µ*m in diameter at the base. Tissues were defined with electrical conductivities taken from literature [46–49], summarized in table 1. We modeled a saline medium external to the anatomical model and set the exterior faces of that medium as the 0 V boundary condition for the electrical simulation. Stimulation was applied by driving a 1 mA current through the exposed contact surface to solve for the extracellular potential throughout the DRG for each electrode (figures 1(b), (c)). During the simulation, this current was scaled by −1 and the desired stimulation amplitude in order to model a cathodic-leading stimulation pulse of the desired amplitude.

**Table 1.** Electrical conductivity values of tissues in FEM, drawn from literature.

Parameters	Values (S m^−1^)	References
Gray matter	0.23	[46]
DRG (Longitudinal)	0.6	[46]
DRG (Transverse)	0.083	[46]
Epineurium	0.6	[47]
Bone	0.02	[48]
Extraneural tissue	0.25	[46]
Encapsulation	0.17	[49]
Platinum	9.4 × 10^6^	

### 2.2. Equivalent circuit neuron models

We created equivalent circuit models of pseudounipolar neurons in the NEURON simulation environment [50]. To evaluate the recruitment properties of muscle-innervating and skin-receptor-innervating neuron types we created models of *Aα* (large diameter fiber) and *Aβ* (medium diameter fiber) neurons. Whereas earlier modeling work [51] characterizing the recruitment of sensory afferents by DRG stimulation focused only on afferent axons, more recent work has explicitly modeled the pseudounipolar structure of DRG neurons to characterize stimulation for the management of chronic pain [52, 53]. This study builds on these neuron models, and represents models consisting of a soma, an AIS, a stem axon terminating in a t-junction, and peripheral and dorsal root axons bifurcating from the t-junction (figure 2(a)). To ensure the densities of cell bodies and axons were consistent with histology, we also added axons of passage throughout the model. Each of the sections noted above has some distinct electrical and morphological features that we describe in the following subsections. Key electrical parameters of the neuron model are summarized in table 2.

**Table 2.** Key electrical parameters of the neuron model.

Parameters	Values
*Electrical parameters*	

Axoplasmic resistivity	70 Ohm*cm
Specific Membrane Capacitance	2 *μ*F cm^−2^ (scaled by diameter for internodal segments)

*Internodal parameters*	

Myelin conductance, per lamella	0.001 S cm^−2^
Myelin capacitance, per lamella	0.1 *μ*F cm^−2^

*Soma parameters*	

Fast Sodium (Na_f_)	0.45 S cm^−2^
Persistent Sodium (Na_p_)	0.0015 S cm^−2^

*Initial segment parameters*	

Fast Sodium (Na_f_) (Proximal)	1.5 S cm^−2^
Persistent Sodium (Na_p_) (Proximal)	0.005 S cm^−2^
Fast Sodium (Na_f_) (Intermediate)	0.90 S cm^−2^
Persistent Sodium (Na_p_) (Intermediate)	0.003 S cm^−2^

*Nodal parameters*	

Fast Sodium (Na_f_)	3 S cm^−2^
Persistent Sodium (Na_p_)	0.01 S cm^−2^
Fast Potassium (K_f_)	0.02737 S cm^−2^
Slow Potassium (K_s_)	0.04106 S cm^−2^
Leak (L_k_)	0.008 S cm^−2^

### 2.3. Axons (dorsal root, peripheral, and axons of passage)

We defined pseudounipolar neuron models with dorsal root and peripheral axons each consisting of 50 1 *µ*m nodes of Ranvier separated by internodal segments. We also instantiated axons of passage consisting of 100 1 *µ*m nodes of Ranvier separated by internodal segments (with no stem axon or cell body). These axons are based on the mammalian motor axon model developed by McIntyre, Richardson, and Grill [54] (the MRG model, see figure 2(a)), which is defined for a discrete set of fiber diameters. To more accurately represent a continuous distribution of cell sizes, we applied curve-fitting to identify the relationship of model properties, such as conductance, internodal distance, and number of myelin lamella, with axon diameter and instantiated neuron models of varying axon diameters (6 *µ*m to 20 *µ*m) using the calculated values. We drew axon diameters from a continuous realistic distribution of neuron fiber diameters present in the DRG (figure 2(c)), derived by applying curve-fitting to values reported by Lloyd and Chang [55]. To account for the systematic difference in diameter between dorsal root and peripheral axons, the diameter of the dorsal root axon is scaled linearly relative to the diameter of the peripheral axon at a factor of 0.87 (figure 2(b)) [56].

The MRG model on which our axon models are based is a double cable model of a mammalian motor axon that contains nodes of Ranvier separated by ten internodal segments with an explicit representation for the myelin attachment segment, paranodal main segment and internode segment. Nodes in the MRG model include fast and persistent sodium (Na_f_, Na_p_ respectively), slow potassium (K_s_) and linear leakage (L_k_) conductances in parallel with the nodal capacitance as shown in figure 2(a). Internodal conductance was represented by a single conductance value (G_i_) in parallel with the membrane capacitance. Ion channel modifications were made to replicate a sensory axon [57], following prior sensory axon modeling work [52, 58]. Specifically, fast potassium (K_f_) channels were added to the nodal and internodal compartments and slow potassium (K_s_), leak (L_k_) and hyperpolarization activated cyclic-nucleotide gated channels were added to the internodal compartments. Additionally, similarly to a previous sensory neuron model [52] the nodal L_k_ was increased from 6 to 8 mS cm^−2^ to reduce membrane hyperpolarization at simulation onset. The *A* parameter of the K_s_ *β* rate constant was increased to 0.06 to better fit experimental values of afterhyperpolarization (AHP) amplitude and duration as has been described in previous studies [52, 53].

### 2.4. Soma

The somata of our pseudounipolar neuron models are defined similarly to a large axon node, whose diameter and length are scaled linearly with peripheral axon fiber diameter (figure 2(b)) by a factor of 2.78, derived from prior data relating the sizes of somata and axons in feline DRG [56]. Freeze fracture studies of the DRG soma and AIS describe the soma and AIS as having the same active channels as the nodes, differing only in the sodium channel densities [59]. Based on these freeze fracture studies, for our model, the Na_f_ and Na_p_ channel densities at the soma were modified to 300 channels *µ*m^−2^ (from the 2000 channels *µ*m^−2^ present in the axon nodes).

### 2.5. AIS

We defined the AIS in three parts. Based on the freeze fracture study noted above [59], we defined a 6 *µ*m proximal section and a 194 *µ*m intermediate section. The Na_f_ and Na_p_ channel densities were 1000 channels *µ*m^−2^ at the proximal section and 600 channels *µ*m^−2^ at the intermediate section of the AIS [59]. Finally, we defined a distal section of the AIS with the same channel density as a node (2000 channels *µ*m^−2^) and a length of 1 *µ*m. This distal section is equivalent to what has been termed the ‘heminode’ in prior literature [42, 52, 53, 60, 61] but due to its continuity with the rest of the AIS (it is not separated by any myelin and is distinguished only by a change in channel density), we have defined it here as the distal AIS.

### 2.6. Stem axon

We defined the stem axon similarly to other axons. However, the stem axon has been observed to display a gradual increase in myelination and an unusual set of internodal lengths relative to the other axons [42, 60]. Therefore, we parametrized four internodal regions along our model’s stem axon with a myelin thickness and internodal length to replicate parametrization used in prior models [42, 52], resulting in a fixed stem axon length of 784 *µ*m (including AIS) across neuron sizes.

### 2.7. Neuron validation

We validated our pseudounipolar neuron models by comparing the action potential shapes of *Aα* and *Aβ* neurons with experimental data [62–67] (table 3). Specifically, we measured AP amplitude in the soma and axon of an *Aβ* neuron (7.3 *μ*m) and an *Aα* neuron (16.0 *μ*m) (sizes chosen to correspond with a prior similar model [52]), as well as the AP duration and AHP magnitude for the somatic AP. Finally, we computed the conduction velocity (CV) by applying intracellular stimulation to the axon and measuring AP time at two points along the axon. Almost all AP parameters fell within the expected range, with the exception of the *Aβ* soma AHP magnitude and the *Aα* CV, both of which fell slightly outside the range. We further validated the range of neuron sizes by examining axon CV and recruitment threshold trends. During validation, CV was found to vary linearly with peripheral axon diameter by a factor of 5.29, which falls within a reasonable range of scaling factors (supplemental figure 1(a)) [67], and recruitment thresholds were inversely related with axon diameter (supplemental figure 1(b)). We also conducted a sensitivity analysis to evaluate the impact of the AIS channel density on recruitment thresholds. Consistent across 10 randomly selected neurons, we found that recruitment threshold in response to epineural stimulation gradually and steadily decreases as channel densities in each segment increase, up to the point where both proximal and intermediate AIS exceed about 800 channels *μ*m^−2^, at which point we see unrealistic spontaneous firing (supplemental figure 2).

**Table 3.** Action potential validation parameters (*marks a parameter value that is outside of the literature range).

Parameters	Value	Literature ranges	Reference
*Aβ* (7.3 *µ*m fiber)			

Soma AP amplitude	118.62 mV	109.72 ± 11.21 mV	[48, 62]
Soma AP duration	0.71 ms	1.29 ± 0.59ms	[48, 63]
Soma AHP magnitude	3.48 mV*	7.9 ± 4.2mV	[48, 63]
Axon AP amplitude	102.59 mV	109.72 ± 11.21 mV	[42, 62, 64]
Conduction velocity	33.09 m s^−1^	18.87 ± 16.32 m s^−1^	[42, 66]

*Aα* (16.0 *µ*m fiber)			

Soma AP amplitude	118.86 mV	109.72 ± 11.21 mV	[49, 62]
Soma AP duration	0.71 ms	0.98 ± 0.2 ms	[49, 63]
Soma AHP magnitude	3.36 mV	6.5 ± 4.2 mV	[49, 65]
Axon AP amplitude	103.71 mV	109.72 ± 11.21 mV	[42, 62, 64]
Conduction velocity	81.95 m s^−1^*	89.7 ± 7.6 m s^−1^	[49, 67]

### 2.8. Neuron placement

We developed a custom three-dimensional packing algorithm that was used to place pseudounipolar neurons according to three different experimental configurations to create the three models that we used in this study. We will refer to these as the realistic model, the random model, and the axon-only model. Neurons in the realistic model were placed to replicate the spatial distribution reported in the literature [44, 45] (figure 2(d)), while neurons in the random model were placed randomly without any constraints on spatial distribution to assess the importance of neuron spatial configuration on recruitment properties, and the axon-only model was populated with axons (no pseudounipolar neurons) to assess the effect of pseudounipolar morphology on recruitment properties. Simplified illustrations of all three models are shown in figure 2(e).

To populate the realistic model, for each neuron, our algorithm allocated a 10% chance that its cell body would be randomly placed anywhere within the DRG volume, to account for the fact that the distribution of cell bodies observed in feline histological cross sections includes some cell bodies within the interior of the structure [44] (these interior cell bodies are not reflected in figure 2(e) for simplicity). In the remaining 90% of cases, we implemented the following procedure to concentrate the majority of cell bodies in the dense cell body–rich region at the exterior of the structure: a random angle (relative to the center of the DRG) was chosen between −30° and 210° as well as a random coordinate (*x*) along the longitudinal direction within 2000 *µ*m from the midpoint of the DRG. If the selected angle was between 30° and 150°, a cutoff radius was set to 2/3 of the total radius of the DRG cross-section at that longitudinal point *x*, otherwise the cutoff radius was set to 4/5 of the total radius of the DRG cross-section at that longitudinal point *x*. A coordinate point between the cutoff radius and the overall radius was then randomly chosen for the location of the cell body. All neurons were placed with cell bodies oriented toward the outer surface of the DRG and stem axons oriented toward the center (±10 degrees), and we performed checks during placement to avoid collisions between neurons. Peripheral and dorsal root axons were concentrated near the center of the structure and ran parallel to the peripheral–central axis of the DRG, with additional axons of passage placed throughout the DRG structure to replicate realistic distributions of axons and cell bodies present in different regions of the DRG.

To populate the random model, our algorithm placed neurons by randomly selecting coordinates within the DRG volume and generating a random angle of orientation for the stem axon, rejecting placements that resulted in any part of the cell being placed outside the DRG volume or collisions with previously placed neurons. Peripheral and dorsal root axons ran parallel to the peripheral–central axis of the DRG, with additional axons of passage placed randomly throughout the DRG structure.

Finally, the axon-only model was populated only with axons of passage, which we placed by selecting random coordinates within the DRG structure. Placements were rejected if they resulted in a collision with a previously placed axon.

### 2.9. Simulations

We conducted simulations using the NEURON simulation environment version 7.7, at a 5 *μ*s time step, for a 10 ms long simulation. Using the FEM model, we calculated the extracellular potential at the location of each node for each neuron in the simulation at a 1 mA stimulation, and scaled it by the desired amplitude of each stimulation pulse. The extracellular potentials calculated in this way were then applied to the nodes in NEURON to assess cellular response to stimulation. To simulate DRG stimulation similar to what was applied in the previous feline study [40], we modeled single biphasic, charge-balanced, cathodic-leading pulses with a pulse duration of 80 *μ*s for the leading cathodic pulse and the anodic pulse immediately following at half the amplitude and twice the duration, and conducted binary searches on stimulation amplitude to determine the recruitment thresholds of neurons in the DRG in response to stimulation via epineural and penetrating electrodes. We also assessed which segments of the neurons (AIS, stem axon, t-junction, or axon) were activated first in response to stimulation via each of the two electrode types. We further compared the dynamic ranges of the two electrodes. Finally, we compared recruitment patterns of *Aβ* and *Aα* afferents to assess differences in sensory modality.

Each model was populated with between 10 000 and 20 000 simulated neurons. In order to model variability in neural responses, we used the following subsampling method (which we performed after running the simulation for all neurons in each model to reduce computational demand by sampling from simulation results rather than running numerous computationally expensive simulations): for each model and electrode type, we randomly sampled 2500 of the simulated neurons in the model. We repeated this random sampling 100 times and used these random samples to conduct our analysis. One adjustment to the subsampling had to be made to allow meaningful comparisons between different models. Our realistic model is set up such that most neurons have parts (primarily cell bodies) near the epineural electrode as well as parts (primarily axons) near the penetrating electrode, based on their positioning within the DRG consistent with past empirical findings [44, 45]. The random model is also likely to have parts near both electrodes due to their pseudounipolar structure including cell bodies and stem axons as well as peripheral and dorsal root axons. However, because the axon-only model does not have any pseudounipolar neurons, its cells will have parts near at most one electrode. This presents a challenge for comparing across models, because sampling an equivalent number of neurons from the axon-only model results in a ‘sparser’ sampling—i.e. far fewer cells in the vicinity of each electrode compared to the realistic or even random models. To make more meaningful comparisons possible, we constrained the volume of the DRG from which we sampled cells in the axon-only model. For the simulation with the penetrating electrode, we drew random samples from a volume of the axon-only DRG model around the tip of the penetrating electrode corresponding to the inner region of densely packed axons in the realistic model (within a 800 *µ*m radius of the DRG center, corresponding to approximately 7700 axons). For the epineural electrode simulation, we drew random samples from a region of the top portion of the DRG with an equivalent cross-sectional area to the region we drew from for the penetrating simulation (>537 *µ*m above the center line of the DRG, corresponding to approximately 6400 neurons). The regions that we drew random samples from are separated by dashed lines in figure 2(e). This ensured that a similar number of neurons had neural elements near the relevant electrode in the subsampled instantiations of each model.

## 3. Results

### 3.1. Pseudounipolar neuron activation sites differ between electrode types

To probe the underlying drivers of electrode selectivity, we compared the sites of activation for neurons that respond at low stimulation amplitudes in each model. Because cell bodies are clustered near the epineural surface of the DRG, we would expect to see that, in the realistic DRG model, epineural electrodes would primarily activate the AIS (action potentials are infrequently initiated in cell bodies [68, 69]), while penetrating electrodes would primarily activate t-junctions and axons. Alternatively, we expected to observe a more random pattern of recruitment in the random DRG model.

Four possible sites of activation were considered for pseudounipolar neurons: the AIS, the stem axon, the t-junction, and the pseudounipolar axons (both dorsal root and peripheral axons were included in this category). No spikes were initiated in cell bodies; however, the AIS directly adjacent to the cell body was a common site of activation. Activated axons of passage were included as a fifth category.

**Figure 3.**
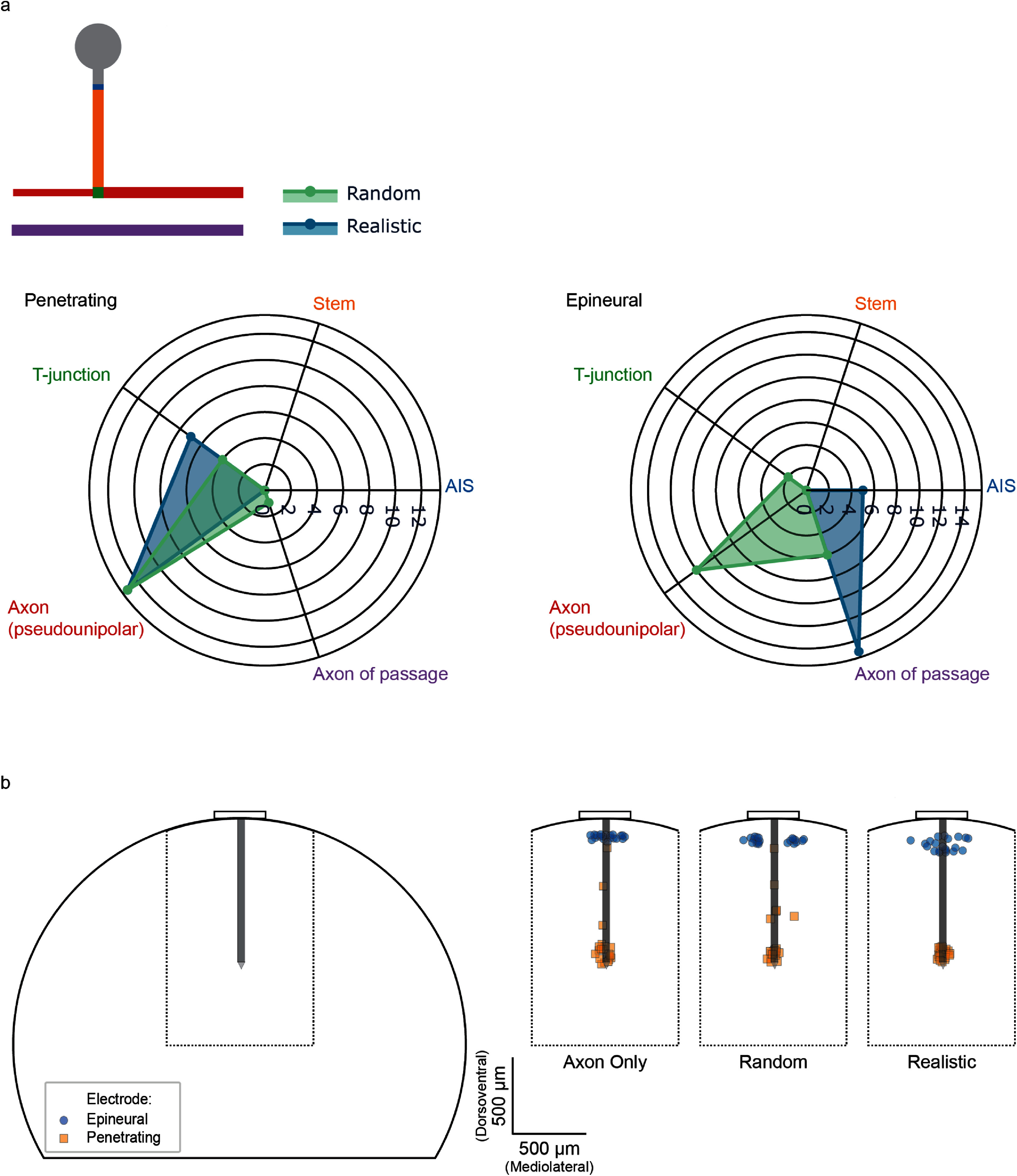
(a) Spike initiation sites for low-threshold neurons in response to penetrating and epineural stimulation. Spike initiation sites differ between random and realistic configurations in response to epineural stimulation but are very similar in response to penetrating stimulation. (Neuron illustration not to scale.) (b) Spatial locations of spike initiation in response to epineural and penetrating electrodes tend to cluster near electrode active sites.

To enable comparison of low-threshold responses across electrode types and models, we selected the first 20 cells activated in response to each electrode in each model (i.e. the 20 neurons with the lowest recruitment thresholds) for each random subsample. Figure 3(a) shows the median number of cells activated at each site of activation for neurons in the random and realistic models in response to stimulation via penetrating and epineural electrodes. For penetrating electrodes, the sites of activation are highly similar for the random and realistic DRG models. Conversely, epineural electrodes in the realistic model primarily activate pseudounipolar neurons at the AIS, as well as nearby axons of passage, whereas they activate a wider array of neuron segments in the random model. The same pattern is seen for the first 10, 20, 50, 100, 200, and 500 neurons that are activated by stimulation (supplemental figure 3). This result suggests that epineural electrodes are more sensitive than penetrating electrodes to the spatial organization of the DRG, and that the clustering of cell bodies near the outer circumference of the DRG makes the AIS of pseudounipolar neurons a more likely target for epineural electrodes.

To quantify the similarity of recruitment across different models, we define a similarity score as follows: we first represent each model-electrode combination as a vector ***N^M,E^***, where *M* is the model, *E* is the electrode, and each *N_i_* represents the number of neurons in model *M* for which spike was initiated at segment *i* (*I* ε {AIS, stem, t-junction, pseudounipolar axon, axon of passage}) is response to electrode *E*. Then the similarity between models for a given electrode *E* is given by Similarity*^E^* or *S^E^* defined as:

\begin{equation*}{ }{S^E}\left( {{\text{model}}1,{\text{ model}}2} \right) = {R^2}\left( {{{\boldsymbol{N}}^{{\boldsymbol{model}}1}},{ }{{\boldsymbol{N}}^{{\boldsymbol{mode}}{\boldsymbol{l}}2}}} \right).\end{equation*}

In other words, *S^E^* can be obtained by plotting the vectors from two different models against each other, in this case the random model vector ***N*^random^** against the realistic model vector ***N*^realistic^**, and computing a linear regression of the resulting line. An *S^E^* value of 1 represents perfect similarity between the recruited segment of the two models. The epineural electrode showed little similarity between random and realistic models (*S*^epineural^ = −0.633) while the penetrating electrode showed strong similarity between the two models (*S*^penetrating^ = 0.934) (calculated using the median ***N^M,E^*** vector over 100 subsamples). To visualize the peri-threshold responses of neurons, we plotted (figure 3(b)) the sites of activation for the first 20 neurons activated in each model by each electrode.

### 3.2. Electrode thresholds are greater for epineural than penetrating electrodes

Electrode thresholds were calculated by determining the minimum stimulation amplitude at which one neuron is activated for each electrode type (epineural and penetrating) and model (axon only, random, and realistic). Electrode threshold values are shown in table 4. We used this measure to compare model thresholds for recruiting a single nerve branch to thresholds reported in our prior empirical work [40].

**Table 4.** Overall electrode thresholds from all cells in the computational model (left), median electrode thresholds across all 100 subsamples of 2500 cells (middle), and median thresholds for selective activation of distal nerve branches in the empirical study in the feline model (right). Threshold magnitudes differ substantially between the computational model and the empirical study. However, when considering the magnitude difference between epineural and penetrating electrode thresholds, both the computational model and the feline model display a similar order of magnitude difference between the electrode types.

	Overall electrode threshold (*μ*A)		Median electrode threshold across all 100 subsamples (*μ*A)		Feline median selective threshold (*μ*A)	
	Epineural	Penetrating	Epineural	Penetrating	Epineural	Penetrating
Axon only	21.15	1.45	21.52 (21.15, 21.82)	3.92 (2.66, 5.85)	—	—
Random	26.04	1.33	28.38 (26.04, 30.98)	1.60 (1.33, 2.45)	—	—
Realistic	22.36	1.16	28.53 (25.10, 31.76)	2.56 (1.16, 3.59)	117.93	10.98

**Figure 4.**
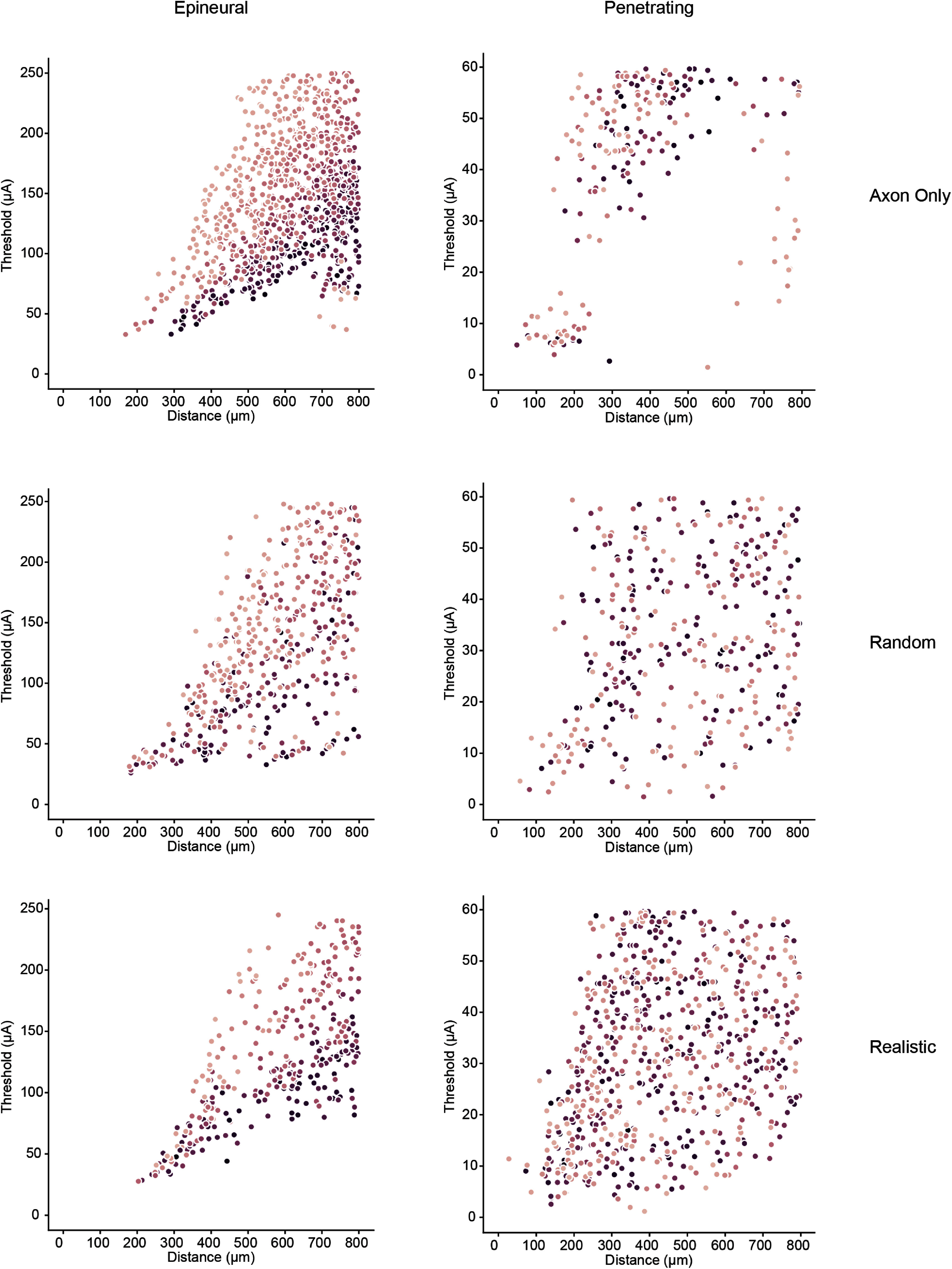
Recruitment threshold as a function of distance from the electrode contact. Darker shades of purple indicate larger fiber diameters.

Consistent with our prior empirical findings [40], we found that in all models, stimulation thresholds for epineural electrodes were much higher than for penetrating electrodes. Table 4 shows the overall electrode threshold for each electrode in each model type on the left, the median electrode threshold with interquartile range across 100 random subsamples for each electrode in each model type in the middle (see supplemental figure 4 for electrode thresholds in all subsamples; median is reported based on a Shapiro–Wilks test for normality which found that thresholds are not normally distributed), and the median threshold for selective recruitment by each electrode in the empirical study in the feline model on the right. Because the metrics for determining threshold differ between the two studies (activation of a single neuron in the computational model vs. activation of a single peripheral nerve branch in the feline model), the threshold values are substantially different between the two. However, when considering the difference in thresholds between the epineural and penetrating electrode, both the computational model and the feline model reveal an approximate 10-fold difference in thresholds between the two electrode types. Within electrode type, the differences in threshold values between models appear to be driven primarily by electrode-to-neuron distance, which we defined as the distance between the center point of the surface of the active electrode and the center point of the activated neuron segment. For epineural electrodes, threshold differences may also be attributable to neuron size/ fiber diameter. The relationship between electrode-to-neuron distance, neuron size, and recruitment threshold is shown in figure 4, which illustrates a distinct relationship between these factors in response to epineural electrode stimulation. Thresholds for penetrating electrode stimulation, on the other hand, are mostly affected by distance from the electrode and far less sensitive to fiber diameter. Critically, this result suggests that the presence of realistic pseudounipolar neuron morphology and DRG spatial configuration does not substantially impact the threshold of activation, but the choice of electrode does.

### 3.3. Epineural electrodes have greater dynamic range than penetrating electrodes

**Figure 5.**
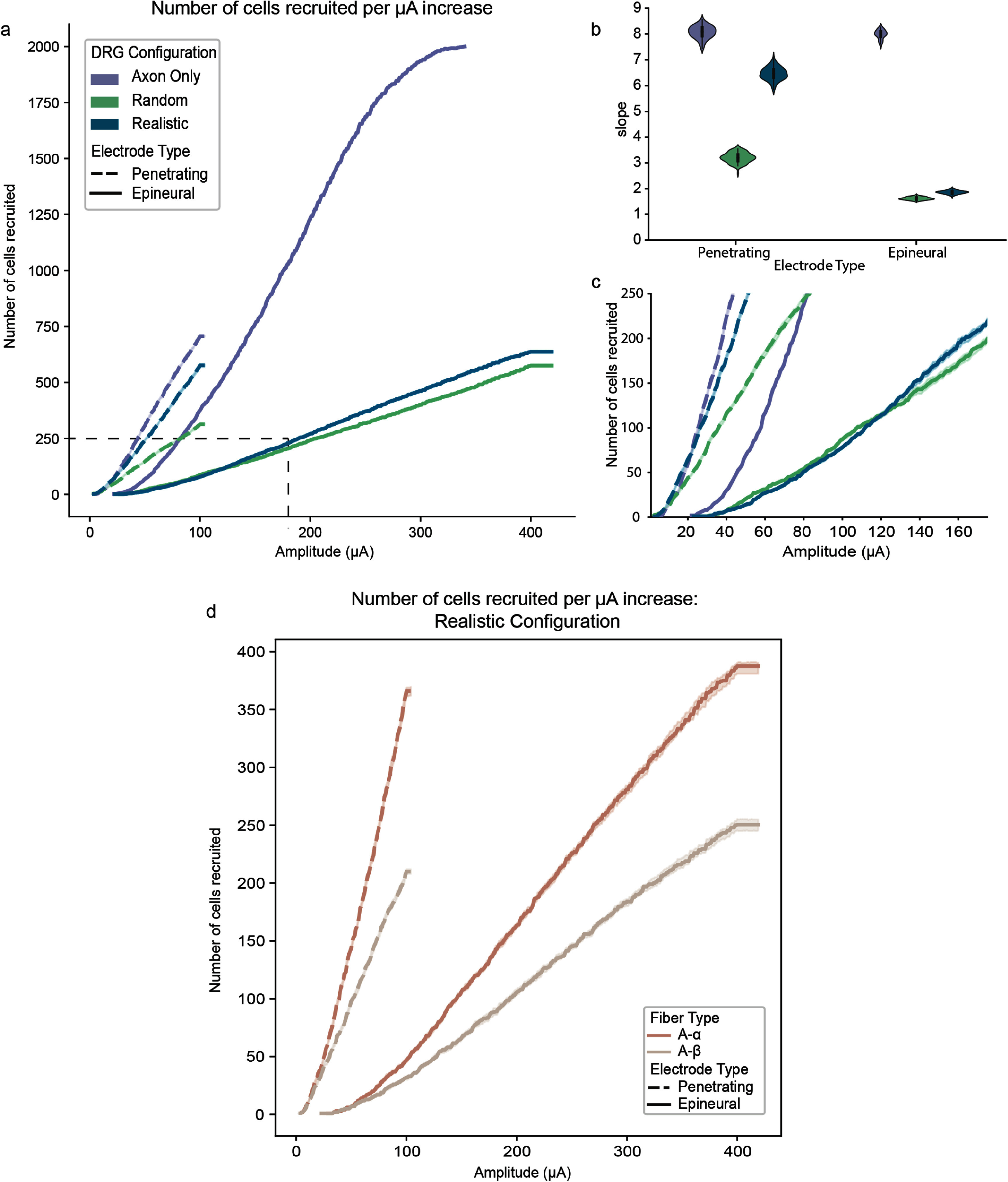
Median recruitment curves (a) and regression line slopes (b) across 100 subsamples for the three DRG models. Epineural electrodes in models containing pseudounipolar neurons (random and realistic) display much wider dynamic range (represented by lower regression line slope) than penetrating electrodes in the random and realistic DRG models. (c) Low-threshold responses follow the same trends seen in panel (a) (plot corresponds to dashed lines in (a)). (d) Recruitment curves comparing *Aα* and *Aβ* neuron recruitment in response to stimulation by epineural and penetrating electrodes. Recruitment curves show little separation, indicating low separability of neuron types.

Our prior empirical study showed that DRG stimulation with epineural macroelectrodes had a greater dynamic range than stimulation with penetrating microelectrodes. A larger dynamic range suggests that a wider set of stimulation amplitudes will produce similar selective responses without off-target side effects, which is an important feature for clinical stimulation applications. To quantify dynamic range in our model, we created recruitment curves displaying the number of additional neurons responding for each 1 *μ*A increase in stimulation above the electrode threshold. Because the curves are very shallow, with a long linear segment, we represent the dynamic range using the slope of the regression line fit to the linear part of each curve, with smaller slopes representing wider dynamic ranges. Median slopes of recruitment curves across the 100 subsamples are listed in table 5. Figure 5(a) shows recruitment curves for each electrode in each model, and figure 5(b) shows the slopes of their regression lines. Consistent with empirical findings, the dynamic range for the epineural electrode is wider than for the penetrating electrode in all models (Kruskal–Wallis test: *p* ≪ 0.001 for each model), although the difference is most pronounced for the realistic model.

**Table 5.** Median slopes (across 100 subsamples) of regression lines fit to linear segment of recruitment curves. Lower slopes correspond to wider dynamic range.

	Axon only model	Random model	Realistic model
Epineural electrode	8.00	1.62	1.87
Penetrating electrode	8.09	3.20	6.48

Comparing models within electrode type, we can see that the dynamic range of a penetrating electrode in a DRG with realistic neuron geometries is similar to that of a DRG containing only axons. This result is consistent with the finding that penetrating electrodes primarily recruit the t-junctions and axons of pseudounipolar neurons in the realistic model where axons are clustered near the center. The dynamic range of an epineural electrode in the realistic model is more similar to that of the random model, suggesting that the pseudounipolar morphology of the DRG neurons drives the larger dynamic range of epineural electrodes as compared to penetrating electrodes. Specifically, the pseudounipolar morphology includes large cell bodies, meaning that each volume of tissue within the DRG can contain fewer neurons than an equivalent volume made up of only axons. In the case of the realistic DRG model, a volume near the center (near the active contact of the penetrating electrode) which is dominated by axons may contain a similar number of neurons compared to the axon-only model, while a volume near the exterior (near the active contact on the epineural electrode) which is dominated by large cell bodies is likely to contain fewer neurons than an equivalent volume of the axon-only model. Supplemental figure 5 shows the dynamic range on a log scale of stimulation amplitude. While the difference in absolute amplitudes between penetrating and epineural stimulation makes log-scale comparisons between them challenging, we can see that within the penetrating electrode condition, the realistic model tracks closely with the axon-only model, while in the epineural electrode condition, the realistic model recruitment curve is nearly identical to the random model and shows a distinctly shallower recruitment than the axon-only model. Figure 5(c) shows the dynamic range for lower stimulation amplitudes (ranging up to 180 *μ*A—inset from dashed lines in figure 5(a)), and illustrates that although the number of neurons activated is nearly equivalent in all models up to a 20 *μ*A stimulation by a penetrating electrode, even at low stimulation amplitudes the dynamic range of the penetrating electrode in the realistic model tracks closely with the dynamic range of the same electrode in the axon-only model, whereas the dynamic range of the epineural electrode in the realistic model tracks closely with the dynamic range of the same electrode in the random model. Moreover, even at low stimulation amplitudes, the epineural electrode in the realistic model exhibits a much wider dynamic range than the penetrating electrode in the same model. This is important to note because evidence from microneurography studies suggests that humans can perceive activity in a single tactile fiber [70], so the ability of epineural electrodes to gradually increase the number of neurons recruited, especially at stimulation amplitudes close to electrode threshold, can be useful for practical applications. Currently available stimulators for clinical use have current steps that range between 1 – 100 *μ*A [71, 72]. At the coarse end of this spectrum (100 *μ*A), the penetrating electrode (in the realistic model, which is closest to actual anatomy) would recruit all possible neurons in one current step, while the epineural electrode would need four current steps to recruit all possible neurons. At the finer end of the spectrum, a stimulator with a 1 *μ*A step could achieve about 100 potentially differentiable steps with a penetrating electrode, compared to 400 potentially differentiable steps with an epineural electrode.

### 3.4. Recruitment of neuron types does not differ sufficiently for sensory modality targeting

To explore the possibility of targeting specific sensory modalities (e.g. for cutaneous vs. proprioceptive feedback) via DRG stimulation, we populated the DRG models with neurons representing both *Aα* and *Aβ* neuron types. Prior empirical and computational modeling work has suggested that despite the larger fiber diameters of *Aα* neurons, they may not be preferentially recruited by electrical stimulation in the DRG [32, 51]. Our models support this conclusion: as shown in figure 5(d), while *Aα* neurons are recruited in slightly higher numbers at all stimulation amplitudes compared to *Aβ* neurons in response to both types of stimulation, the recruitment curves do not indicate any stimulation amplitude where they can be preferentially recruited to the exclusion of *Aβ* neurons.

### 3.5. Electrode location affects epineural electrodes less than penetrating electrodes

**Figure 6.**
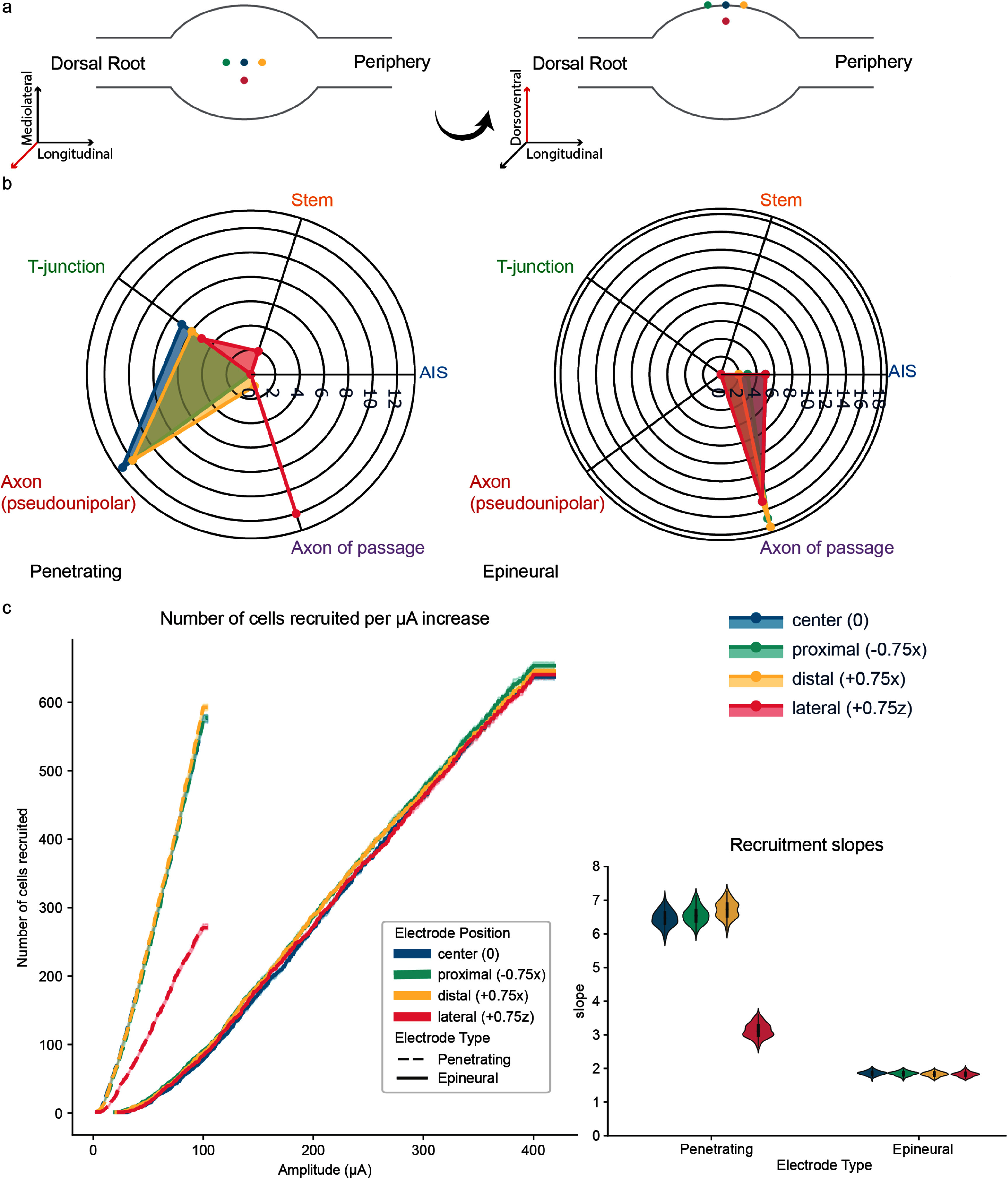
(a) Electrode placements on the dorsal surface of the DRG, with color key (DRG outline not to scale). (b) Spike initiation segments recruited by epineural and penetrating electrodes at the center of the dorsal surface and shifted longitudinally and laterally. Recruitment pattern of the epineural electrode is stable across spatial shifts, whereas the laterally shifted penetrating electrode displays a different recruitment profile. (c) Recruitment curves and regression slopes for the shifted electrodes. The epineural electrode displays relative stability of dynamic range across spatial locations, while the laterally shifted penetrating electrode again shows a different profile relative to other penetrating electrode placements.

To assess the effects of changing electrode location on DRG neuron recruitment, we tested four different electrode locations: the center of the dorsal DRG surface (‘0’), shifted distally by 0.75 mm (‘+0.75x’), shifted proximally by −0.75 mm (‘−0.75x’), and shifted laterally by 0.75 mm (‘+0.75z’) (see figure 6(a)). Because the DRG models are not systematically asymmetric on the medio-lateral axis, the laterally shifted electrode was only shifted in the positive direction. Comparing within electrode type, the first 20 neurons activated by epineural stimulation at all 3 shifted electrode positions are recruited at a very similar set of segments to the centered electrode (figure 6(b)). Using the similarity score *S^E^* metric that was previously described, we can compare each shifted electrode with the center electrode and find *S^E^* values. Overall we see high similarity across electrode shifts in both electrodes, with a pronounced difference in the laterally shifted penetrating electrode. *S^E^* values for the first 20 activated neurons (calculated using the median ***N^M,E^*** vectors over 100 subsamples) are as follows: *S*^epineural^(0, +0.75x) = 0.842, *S*^epineural^ (0, −0.75x) = 0.930, *S*^epineural^ (0, +0.75z) = 1.0, *S*^penetrating^ (0, +0.75x) = 0.980, *S*^penetrating^ (0, −0.75x) = 0.987, *r*^2^ (+0.75z) = −1.121. These trends are borne out for the first 10, 20, 50, 100, 200, and 500 neurons activated (supplemental figure 6). The laterally shifted penetrating electrode displays a marked difference compared to the others. This is likely due to the distinct arrangement of neurons in the realistic model. The orientation of the shifted electrode is the same as that of the original, centrally placed, electrode. However, because the electrode has been shifted by 750 *µ*m toward the lateral part of the DRG (half the DRG diameter), the surface of the active electrode contact is positioned in a region that is far less densely packed with pseudounipolar axons than the center of the DRG is. It is instead in a region that is largely populated with axons of passage and the stem axons connecting the neurons whose cell bodies are placed in the lateral part of the DRG with their pseudounipolar axons in the interior, as reflected by the sites of action potential initiation that prevail in this condition.

We also compared the dynamic range exhibited by each shifted electrode with the center electrode. Dynamic range values were similar across shifted electrodes, with the laterally shifted penetrating electrode again displaying a noticeable difference from the others (figure 6(c)).

## 4. Discussion

In this work we sought to explore the mechanisms of selective neural activation in response to epineural DRG stimulation, an understudied approach to the delivery of sensory feedback that can leverage an existing clinical technology. To that end, we developed the models in this paper with the goal of understanding the roles of DRG pseudounipolar neuron morphology and DRG spatial organization on the selective activation of neural structures in response to DRG stimulation by penetrating and epineural electrodes. We show that the behavior of the model aligns with empirical data from prior feline experiments in that epineural macroelectrodes display a higher electrode threshold and greater dynamic range compared with penetrating microelectrodes. By comparing three models (axon only, random, and realistic), we can identify some of the mechanisms that underlie selective activation of nerve branches by epineural DRG stimulation.

We posit that the spatial organization of the DRG [44, 45] enables selective activation by epineural electrodes. Specifically, the concentration of cell bodies near the circumference of the structure might suggest that cell bodies may be more easily recruited by epineural stimulation, rather than axons. However, prior work has shown that cell bodies are infrequently activated directly by extracellular stimulation, with adjacent AIS and/or stem nodes showing initial activation [68, 69]. Our data are consistent with these findings: in neurons recruited by low-amplitude epineural stimulation, spikes are much more likely to be initiated in the AIS, adjacent to the cell body, than in other parts of the pseudounipolar neuron. By contrast, the penetrating electrode is much more likely to initiate activation in the axons of the pseudounipolar neuron than the AIS. Since recruitment thresholds for the AIS tend to be higher than those of nearby axons[69], this does not suggest preferential activation of the AIS over other neuron regions. Rather, the impact of the cell bodies seems tied to the packing density of the neural tissue. Because cell bodies are much larger in diameter than axons, they are less densely packed, meaning that while epineural stimulation may activate a larger volume of tissue, the number of neurons activated does not necessarily scale accordingly. Therefore, despite not being directly activated, the presence of the cell bodies can account for the surprising degree of selectivity that is observed empirically in response to epineural DRG stimulation.

The heightened probability of AIS excitement by epineural electrodes compared with penetrating electrodes is likely a result of the greater concentration of the proximal AIS regions close to the epineural electrode. The AIS is known to modulate polarity and excitability of neurons [73, 74] and acts as the site of action potential initiation in most multipolar neurons [75]. In normal sensory transmission along sensory afferent neurons, action potentials are initiated at the distal end of the peripheral axon, not the AIS [76]. However, as our model has demonstrated, in the case of epineural DRG stimulation, externally applied stimulation can initiate action potentials at the AIS of DRG neurons. Recent work has shown that heightened Na_v_ 1.7 channel expression in the AIS of DRG neurons can lead to spontaneous firing that may be implicated in neuropathic pain [43]. Moreover, both the morphological and electrogenic properties of the AIS can change with age and a variety of morphological factors [77]. In this work, we modeled the AIS based on freeze-fracture studies that characterized the channel density of the AIS of the DRG [59], and did not observe any spontaneous firing. We have shown that action potential initiation at the AIS is possible and that those action potentials can further propagate reliably into the central axon to convey signals into the spinal cord (see overlaid traces in supplemental figure 7). It is possible that prolonged epineural electrical stimulation may alter the properties of the AIS, but future *in vivo* work will be required to explore this possibility. Furthermore, changes to morphological and electrical properties of the modeled AIS including channel density may have a substantial impact on neuronal excitability. To test this, we conducted a sensitivity analysis of the AIS channel density and the relative length of the proximal and intermediate AIS using 10 neurons that were randomly sampled from the set of neurons that showed initial activation at the AIS (supplemental figure 2). We found that increasing channel density generally led to gradually decreasing threshold, except when both proximal and intermediate AIS had channel densities exceeding 800 channels *μ*m^−2^, at which point spontaneous firing was observed. (This is consistent with prior modeling work [52] that used a uniform AIS and adjusted the channel density down from 800 channels *μ*m^−2^ to 500 channels *μ*m^−2^ to avoid spontaneous firing.) This highlights the critical role of the AIS and the need for further study into its mechanisms and role as a site of action potential initiation during extracellular electrical stimulation.

So far we have largely discussed the role of the soma in our results as a function of its size affecting the density of neural tissue in different regions of the DRG. However, the soma may also be influencing neural recruitment by acting as a large axon terminal in the vicinity of the stimulating electrode. Early models of neuronal activation in response to extracellular stimulation showed that due to its large size and high capacitance, the soma is infrequently the site of action potential initiation but that extracellular stimulation near the soma evokes action potentials in the initial segment or first node [68]. Consistent with that earlier finding, we do not observe any direct activation of the soma by stimulation, but instead see substantial action potential initiation at the AIS. However, the soma does appear to exert substantial influence on the electrical environment of the neighboring nodes, even as far away as the t-junction. As the voltage traces in supplemental figure 7 illustrate, the soma membrane depolarizes during the leading, cathodic phase of the stimulation pulse, and hyperpolarizes during the anodic phase. Conversely, the AIS, stem axon nodes, and t-junction all exhibit the inverse response. This suggests that a current flow within these neighboring nodes that is driven by the response of the soma, consistent with prior work that has shown this pattern in response to cathodic stimulation, where ‘the depolarization in the cell body [is] not large enough to generate an action potential, but contribute[s] to the repolarization of the first node after the period of hyperpolarization during the stimulus.’ [69] Therefore, although the soma is never the site of action potential initiation, it does appear to play a role in the excitability and responsivity of its neighboring segments to stimulation.

Since the packing density of neural tissue and the presence of cell bodies near the circumference is a feature of DRG anatomy rather than a feature of the electrode, the advantages that this feature confers on the epineural electrode are not necessarily unique to the epineural electrode. In fact, one could model a penetrating electrode with a short shank such that the active electrode contact could be situated in a cell body-rich region. However, a few considerations diminish the practical effectiveness of this approach. Firstly, in order to situate the active contact in the annular region that is densely packed with cell bodies the penetrating electrode shank would have to be less than 0.5 mm long. This is very small relative to standard penetrating electrodes and would likely present challenges for anchoring the electrode in the tissue, making chronic implants challenging. Moreover, the volume of activated tissue for a penetrating electrode is usually relatively small compared to an epineural electrode. In a less densely packed, cell-body-rich region, this would likely require high stimulation amplitudes from the penetrating electrode to recruit an equivalent number of neurons. Because of the small surface area of the penetrating electrode, this could lead to a heightened risk of electrochemical tissue damage. This study aimed to show how features of the DRG and electrode could enable a practical application of an epineural electrode that can perform as well as a penetrating electrode, and therefore focused on epineural electrodes that are likely to have a better safety profile and more straightforward path to clinical translation than penetrating electrodes with short shanks.

Another area of interest in this study was dynamic range. In our results, the dynamic range of the epineural electrode was wider than that of the penetrating electrode. A wider dynamic range is desirable for a somatosensory neuroprosthesis because it indicates that the recruitment of additional neurons can be separated into a larger number of stimulation amplitude steps, potentially allowing for more graded control over the intensity of evoked sensations. In our models, the difference in dynamic range between epineural and penetrating electrodes varied across different models. In the axon only model, the dynamic range of both electrode types was very similar and small. This result is unsurprising as the electrode contacts have access to similarly densely packed axons. In the random model, we saw a relatively high dynamic range for both electrode types. This result is likely reflective of a uniformly low density (compared to the axon only model) of neurons accessible to both electrode types. The most marked difference in dynamic range between the penetrating and epineural electrodes appeared in the realistic model, where the penetrating microelectrode has access to densely packed axons in the interior of the DRG structure while the epineural macroelectrode has access to larger cell bodies and their AIS at the exterior of the structure. The size of the cell bodies precludes the kind of dense packing that is possible in the DRG interior, therefore enabling a less steep recruitment curve (i.e. a wider dynamic range).

In this model we were only able to quantify dynamic range in terms of the number of neurons activated per unit increase in stimulation amplitude. However, the functional importance of dynamic range is primarily understood in terms of perceptual differentiability. Unfortunately, neither computational nor animal models can provide perceptual results. The feline study compared activation of distal nerve branches but lacked a way to report perceptual differences, while the computational model can only report the number of neurons activated. Therefore, to try and contextualize these results in a meaningful somatosensory paradigm, we must lean on what is known about sensory perception and existing clinical stimulator technology. Microneurography studies have shown that activity in a single tactile fiber can be perceived [70], suggesting that considering dynamic range at the level of single neuron increases in activation could be a perceptually meaningful measure. Using a commercial stimulator device for clinical use, the Digitimer DS8r, which has a step size of 100 *μ*A [71], stimulation delivered by a penetrating electrode would recruit all neurons in our model within two amplitude steps, precluding any possibility of granular perceptual changes. An epineural electrode, on the other hand, would be able to achieve five discrete amplitude steps which we may expect to correspond to five distinct perceptual levels. Another existing system (the Ripple Neuro Nano2 + Stim system), can deliver 100 discrete current steps at a step size of 1, 2, 5, 10, or 20 *μ*A/step [72], which could enable similar granularity of perceptual changes for both electrode types. The ability of this system to deliver similar perceptual granularity in the two different electrodes depends on several factors, including what the maximum perceptually meaningful amplitude of the epineural electrode is and how many additional neurons need to be recruited at each step to achieve a perceptible difference. Naturally these predictions cannot be tested without future work in human participants to quantify the actual perceptual output of such stimulation. However, it is important to note that differences in the step sizes achievable by clinical stimulators, especially as stimulator technology advances, will affect how important the dynamic range is for controlling percept intensity.

We were further interested in the ability of epineural and penetrating electrodes to differentially activate different neuron types. Prior empirical and computational modeling work [32, 51, 53] has shown that DRG stimulation via penetrating microelectrodes preferentially activates medium diameter (*Aβ*) fibers as compared to large diameter (*Aα*) fibers. This may be a result of the relative distribution of these neuron types in the DRG, which has a higher density of *Aβ* than *Aα* neurons. Conversely, other computational modeling work [78] suggests that larger diameter fibers may be more likely to be recruited in response to stimulation, consistent with the expected inverse recruitment order with fiber diameter normally observed in peripheral nerves [79]. Our model explicitly represented fibers of different types in order to investigate whether a DRG somatosensory neuroprosthesis could achieve neuron-type selectivity. The wide dynamic range of the epineural electrode presented an interesting possibility: if the recruitment curves of *Aα* and *Aβ* neurons are separable, this would enable selective recruitment of one neuron type (and potentially one sensory modality) over another. However, our model results reveal that these recruitment curves are not separable for either electrode type, consistent with previous findings [32, 51] that both fiber types are recruited together during DRG stimulation.

Although this work is an advance toward accurate modeling of the DRG, there are still several limitations to the model. Neuron trajectories are simplified, with stem axons rotating in the cross-sectional plane and axons placed in straight trajectories along the central–peripheral axis. This approach does not represent the winding and complicated trajectories that exist in real tissue [41], making up the ‘glomerulus’ of DRG neurons. Moreover, this model does not represent the wrapping that is observed of the stem axon around the cell body in real DRG [41]. A recent modeling study that implemented winding glomerular trajectories (although without wrapping around the cell body) found that glomerular trajectories had an impact on the waveforms recorded from the neurons in response to stimulation [80], suggesting that this could affect recruitment properties. However, while the inclusion of these trajectories may affect recruitment thresholds or sites of action potential initiation, we would not expect a major change in the central finding of this study, as these trajectories would not change the packing density of different regions of the DRG. Another limitation is the difficulty of mapping model results to empirical results from the previous feline study. The empirical study used cuff electrodes to record evoked compound action potentials from peripheral nerves in order to measure selective activation of distal nerve branches to approximate the region of the limb in which the activation would be perceived. It was not, however, possible to quantify how many neurons were included in the evoked responses recorded with this approach. On the other hand, because no existing literature points to a somatotopy of neurons in the DRG, it is not possible to map modeled neurons to distal nerve branches. This discrepancy makes it challenging to make direct comparisons with empirical results or to predict nerve branch-level selectivity from the model. Instead, we focused on quantifying the number of neurons recruited as a measure of selectivity. This also leads to a discrepancy in comparing thresholds between the model and the empirical results. In the computational model we define electrode threshold as the threshold of activation for a single neuron, as there is evidence to suggest that activity in a single tactile neuron is perceptible[`], but this threshold can be subject to random variability in single neuron factors like size and location relative to the electrode. Nevertheless, when comparing orders of magnitude between electrode types in the empirical and simulated data, we see that epineural electrode thresholds exceed penetrating electrode thresholds by a similar order of magnitude in the realistic computational model and in the empirical results. Despite these limitations, our model has demonstrated a principle based on the density of neuronal elements in different regions of the DRG that closely matches results from our prior empirical study.

## 5. Conclusion

This model has expanded on prior sensory neuron models and DRG models in multiple key ways. Firstly, our model has instantiated neurons with a continuous distribution of fiber diameters, including cell bodies that scale with their axons. Secondly, the model is populated with a realistic, data-based distribution of neuron sizes in order to accurately represent the population of neurons within the DRG. Finally, we have implemented a model representing the accurate spatial distribution of neurons within the DRG, in addition to two control models that enable careful consideration of the role of DRG neuron morphology and DRG spatial configuration in neural responses to stimulation at the DRG. Using these models, we have identified a likely mechanism for the selective activation of nerve branches by epineural DRG stimulation as demonstrated in prior work.

We have demonstrated that the unique neuron morphology and spatial organization of the DRG enable an epineural DRG electrode to achieve selective activation and make the DRG a compelling target for electrical stimulation for delivering focal sensory feedback from prosthetic devices. Follow-up trials with human participants will be necessary to determine how best to use DRG stimulation for this application. Future work may also build on this modeling approach to explore the effects of different electrode configurations and stimulation paradigms on DRG stimulation recruitment properties. For example, multipolar epineural electrode configurations may enable current steering to better target specific neuron populations and/or sensory modalities that cannot be accessed by a single electrode with a distant return. Although further work is needed to develop this approach into a clinical somatosensory neuroprosthesis, our model and results indicate that thanks to the morphological features of the DRG, such a future neuroprosthesis may leverage existing clinical epineural DRG electrodes to deliver focal sensory feedback to a user.

## Data Availability

The data that support the findings of this study are openly available at the following URL/DOI: https://github.com/pitt-rnel/DRG-model-data2024. The DRG neuron model can be downloaded at: https://modeldb.science/2018004.
